# CD103 (αE Integrin) Undergoes Endosomal Trafficking in Human Dendritic Cells, but Does Not Mediate Epithelial Adhesion

**DOI:** 10.3389/fimmu.2018.02989

**Published:** 2018-12-21

**Authors:** Steve Swain, Mandi M. Roe, Thomas A. Sebrell, Barkan Sidar, Jennifer Dankoff, Rachel VanAusdol, Lesley E. Smythies, Phillip D. Smith, Diane Bimczok

**Affiliations:** ^1^Department of Microbiology and Immunology, Montana State University, Bozeman, MT, United States; ^2^Chemical and Biological Engineering Department, Montana State University, Bozeman, MT, United States; ^3^Division of Gastroenterology and Hepatology, School of Medicine, University of Alabama at Birmingham, Birmingham, AL, United States

**Keywords:** gastrointestinal epithelium, integrin alpha E, antigen-presenting cell, cell adhesion, endosome

## Abstract

Dendritic cell (DC) expression of CD103, the α subunit of αEβ7 integrin, is thought to enable DC interactions with E-cadherin-expressing gastrointestinal epithelia for improved mucosal immunosurveillance. In the stomach, efficient DC surveillance of the epithelial barrier is crucial for the induction of immune responses to *H. pylori*, the causative agent of peptic ulcers and gastric cancer. However, gastric DCs express only low levels of surface CD103, as we previously showed. We here tested the hypothesis that intracellular pools of CD103 in human gastric DCs can be redistributed to the cell surface for engagement of epithelial cell-expressed E-cadherin to promote DC-epithelial cell adhesion. In support of our hypothesis, immunofluorescence analysis of tissue sections showed that CD103^+^ gastric DCs were preferentially localized within the gastric epithelial layer. Flow cytometry and imaging cytometry revealed that human gastric DCs expressed intracellular CD103, corroborating our previous findings in monocyte-derived DCs (MoDCs). Using confocal microscopy, we show that CD103 was present in endosomal compartments, where CD103 partially co-localized with clathrin, early endosome antigen-1 and Rab11, suggesting that CD103 undergoes endosomal trafficking similar to β1 integrins. Dynamic expression of CD103 on human MoDCs was confirmed by internalization assay. To analyze whether DC-expressed CD103 promotes adhesion to E-cadherin, we performed adhesion and spreading assays on E-cadherin-coated glass slides. In MoDCs generated in the presence of retinoic acid, which express increased CD103, intracellular CD103 significantly redistributed toward the E-cadherin-coated glass surface. However, DCs spreading and adhesion did not differ between E-cadherin-coated slides and slides coated with serum alone. In adhesion assays using E-cadherin-positive HT-29 cells, DC binding was significantly improved by addition of Mn^2+^ and decreased in the presence of EGTA, consistent with the dependence of integrin-based interactions on divalent cations. However, retinoic acid failed to increase DC adhesion, and a CD103 neutralizing antibody was unable to inhibit DC binding to the E-cadherin positive cells. In contrast, a blocking antibody to DC-expressed E-cadherin significantly reduced DC binding to the epithelium. Overall, these data indicate that CD103 engages in DC-epithelial cell interactions upon contact with epithelial E-cadherin, but is not a major driver of DC adhesion to gastrointestinal epithelia.

## Introduction

Dendritic cells (DCs) frequently interact with the epithelial layer of the gastric mucosa, as shown in previous studies (1–3). As professional antigen-presenting cells, DCs control the immune response to *Helicobacter pylori* (*H. pylori*) (4), a bacterial pathogen that causes chronic gastritis, peptic ulcer disease, and gastric cancer (5–7). Specifically, the type of T cell response induced by the DCs largely determines whether *H. pylori* infection causes only mild inflammation or leads to severe inflammatory pathologies including ulcers or cancer (8–10). For those DCs that are located immediately beneath or within the gastric epithelium, their spatial interactions with the epithelial cells have important functional implications for the immune response to *H. pylori*. First, DCs that reside within the epithelial layer or extend transepithelial dendrites have direct access to the gastric lumen for *H. pylori* antigen sampling (1–3, 11). Second, positioning of gastric DCs immediately below the epithelium increases the probability for pathogen capture upon epithelial barrier breach, and third, the close proximity of DCs to epithelial cells likely enhances the paracrine effects of epithelial-derived mediators that regulate DC function (12–14). In spite of the importance of DC-epithelial interactions for gastrointestinal immune responses, the molecular mechanisms of these interactions are not well-defined.

Binding of DC-expressed CD103 (αEß7 integrin) to epithelial E-cadherin was proposed as a potential mechanism for DC adhesion to epithelial cells (15–17). CD103, the α subunit of αEß7 integrin, is widely recognized as an important DC subset and lineage marker in humans and mice (18–20). Specifically, CD103 identifies a DC subset termed conventional DC1 that is able to cross-present exogenous antigens to CD8 T cells and that induces mucosal tolerance to commensals and dietary antigens (18, 21). The functional role of CD103 has been extensively studied in transfected cells lines, where the A-domain of the αE (CD103) integrin subunit was shown to interact with the top surface of E-cadherin domain 1, and in intestinal intraepithelial lymphocytes (IELs), where CD103 anchors the IELs within the epithelial layer (22–24). Inspite of its frequent use as a DC marker, the function of CD103 in primary human DCs has received little investigative attention. Therefore, the goal of our study was to determine whether CD103 enables DCs in the human stomach to interact with the epithelium through E-cadherin engagement.

Notably, previous studies from our laboratory and others have shown that surface CD103 expression of gastric DCs is low compared to CD103 expression on DCs in other tissue compartments, such as the small intestine (14, 25–27). This low surface CD103 expression was unexpected, since gastric DCs have a tolerogenic capacity similar to that of human intestinal DCs (14, 28) and also are efficient producers of retinoic acid (RA), properties generally associated with intestinal CD103^+^ DC subsets (14, 29, 30). However, we also showed that human monocyte-derived DCs express considerable amounts of CD103 in intracellular compartments (26). Other integrins including α5β1, α6β4, and αMβ2 are expressed in endosomal compartments and recirculate through the membrane to enable dynamic and tightly regulated interactions with their respective ligands (31–33). Therefore, we hypothesized that intracellular pools of αE integrin/CD103 present in human gastric DCs can be redistributed to the cell surface for engagement of epithelial cell-expressed E-cadherin in the stomach to promote DC-epithelial cell adhesion. Interestingly, our experiments revealed that CD103 undergoes endosomal trafficking in human DCs and is engaged upon DC contact with epithelial E-cadherin, but is not the major adhesion factor that mediates epithelial cell binding.

## Materials and Methods

### Human Blood and Tissue Samples

Heparinized blood samples were obtained with local IRB approval from healthy adult volunteers in Birmingham, AL (IRB# X120806005), or Bozeman, MT (IRB #DB082817 and #DB092614). Gastric tissue specimens were obtained with Institutional Review Board (IRB) approval and informed consent from non-*H. pylori*-infected adult subjects undergoing elective gastric bypass surgery or sleeve gastrectomy for treatment of obesity at the University of Alabama at Birmingham (IRB# F120815005) or were provided as exempt specimens by the National Disease Research Interchange (Philadelphia, PA; IRB# DB062615-EX).

### Antibodies

The following mouse anti-human monoclonal antibodies were used for flow cytometry, imaging cytometry and confocal analysis of MoDCs: HLA-DR (clone L243), CD11c (B-ly6), CD103/αE (B-Ly7,) CD3 (HIT3a), CD19 (SJ25C1), CD45 (2D1), CD56 (MY31), E-cadherin (67A4), CD49d (9F10) purchased from eBioscience, Biolegend, or Tonbo, all San Diego, CA. Endosomal compartments were labeled with rabbit anti-human clathrin (D3C6, Cell Signaling, Danvers, MA), rabbit anti-human EEA-1 (polyclonal), mouse anti-human Rab7a (Rab7-117), and rabbit anti-human Rab11 (polyclonal), all from Abcam, Cambridge, MA. The following monoclonal antibodies were used for staining of paraffin-embedded tissue sections: anti-human HLA-DR (LN-3) and anti-human CD103 [EPR4166(2)], both Abcam, Cambridge, MA. The following antibodies were used in neutralization assays: anti-human CD103 (2G5) (Beckman Coulter, France) and anti-human E-cadherin (SHE78-7) (Thermo Fisher Scientific, Waltham, MA). Appropriate isotype-matched control antibodies were used in all experiments.

### Dendritic Cells

To obtain human gastric DCs, mucosal tissue was subjected to three rounds of EDTA treatment and then digested with collagenase solution to obtain lamina propria mononuclear cells, as described previously (14, 26). Gastric DCs were then enriched using MACS sorting for HLA-DR^+^ cells (Miltenyi Biotec, Auburn, CA).

To generate monocyte-derived DCs (MoDCs), PBMCs were isolated using Ficoll density gradient centrifugation, and MoDCs were differentiated from MACS-isolated CD14^+^ blood monocytes by culturing 2 × 10^6^ monocytes per well in 24-well plates in complete medium (DMEM, 10% heat-inactivated human AB serum and antibiotics) or serum-free medium (X-Vivo 10, with HEPES and L-Glutamine) supplemented with recombinant human GM-CSF (25 ng/mL), and IL-4 (17 ng/mL), both from R&D Systems, Minneapolis, MN (26, 28). To enhance DC CD103 expression, 100 nM retinoic acid (RA, Sigma, St. Louis, MO) was added to some MoDC cultures, as described previously (26). Cytokines and RA were replenished after 3 days, and after 5–6 days, non-adherent cells were harvested as MoDCs by vigorous pipetting.

### Immunofluorescent Labeling of Tissues and Cells for Microscopy

We used 4 μm paraffin-embedded sections to analyze CD103 expression by human gastric DCs *in situ*. Sections were de-paraffinized and then incubated in a vegetable steamer for 30 min in pre-heated Unmasking Solution (Vector Laboratories, Burlingame, CA) for antigen retrieval. Sections were then blocked in normal goat serum and incubated in the presence of primary antibodies overnight. Species specific secondary antibodies labeled with Alexa 488 or Alexa 555 (SouthernBiotech, Birmingham, AL) were added for 30 min. Finally, nuclei were stained with DAPI, and sections were mounted in Fluoroshield (Sigma-Aldrich) and sealed with nail varnish. For microscopic analysis of MoDCs, cells were stained either directly on glass-bottom plates or chamber slides (CD103 distribution and spreading assays) or were stained in suspension and then spotted onto glass slides (endosomal markers). For intracellular labeling, DCs were permeabilized with Cytofix/Cytoperm solution (Becton Dickinson) for 20 min at 4°C, washed with PermWash buffer (Becton Dickinson) and then were incubated with antibodies for 30 min at 4°C. Nuclei were labeled with DRAQ5 or DAPI.

### Microscopy and Image Analysis

Immunofluorescence analysis of slides was performed on an Olympus BX60 upright fluorescence microscope equipped with a DS-Ri1 digital camera and with NIS Elements software (Nikon, Melville, NY) or on an EVOS FL Cell Imaging system (Thermo Fisher Scientific). Confocal microscopy images were acquired using a Zeiss LSM 510 META system, with a 63 × objective and a step size of 0.5 μm, or an inverted Leica SP5 Confocal Scanning Laser Microscope (Leica, Wetzlar, Germany) with a 20 × objective or a 63 × water immersion objective with Immersol (W 2010, Zeiss, Oberkochen, Germany). Digital image analysis was performed using ImageJ 1.48v software. The distribution of CD103^+^ DCs in relation to the epithelium in gastric tissue sections was determined by manual counting on digital images, using NIS Elements software. Three-dimensional co-localization of red (endosomal markers) and green (CD103) voxels in confocal stacks was determined using the JaCOP plugin to calculate Mander's co-localization coefficient (34). Part of this procedure involved screening 16 algorithms for optimal exclusion of background staining; the Bernsen method of auto-thresholding was chosen and applied objectively to all images (35). In tissue sections, regions of interest were set to exclude surface and glandular epithelial cells.

### FACS Analysis

For flow cytometry, cells were labeled with pre-determined optimum concentrations of antibodies at 4°C for 15 min, followed by washing in FACS Stain Buffer (BD Biosciences). For intracellular staining, cells were fixed and permeabilized with Cytofix/Cytoperm (BD Bioscience), and antibodies we added in the presence of BD PermWash buffer. Dead cells were labeled with LIVE/DEAD® yellow dye (Life Technologies, Carlsbad, CA). A BD LSR or LSRII was used for flow cytometry; and data were analyzed using FlowJo V10 software (Treestar, Ashland, OR). Gastric DCs were gated as live CD45^pos^/lineage^neg^/HLA-DR^high^ cells. The lineage cocktail contained antibodies to CD3, CD19, and CD56.

### Imaging Cytometry

Imaging cytometry was performed using an ImageStreamX Mark II (Amnis Corp., Seattle, WA). Cells were prepared as described for FACS analysis, with 7-AAD or DAPI used to label nuclei in fixed cells. Data were analyzed with IDEAS software v6.1 (Amnis Corp.). The following channels were recorded: Ch 1–brightfield, Ch 2–CD103 FITC, channel 3–CD11c PE, and channel 5–7-AAD or channel 7–DAPI. DCs were gated as focused cells based on Gradient RMS Ch 1, single cells based on Aspect Ratio and Area Ch1, and Intensity of CD11c PE in Ch 3.

### Internalization Assay

Relative rates of internalization of CD103 were determined in MoDCs using a variation of the method from Chen et al. (36). Briefly, aliquots of MoDCs generated in the presence of retinoic acid (100 nM) were chilled to 4°C, then incubated with FITC labeled mouse anti-human CD103 for 30 min. Unbound antibody was then removed by washing in ice cold media. Time zero samples were left on ice, and internalization was initiated in the remaining samples by resuspending the samples in 37°C media and incubating them at that temperature for the indicated times. At each time point, samples were quickly washed in ice cold FACS buffer and left on ice to inhibit further internalization. After washing, cells were incubated for 30 min with anti-mouse IgG eFluor660 to label remaining cell surface anti-CD103 antibodies. Cells were then washed again in cold FACS buffer and analyzed with a BD LSR flow cytometer. Mean fluorescent intensities were normalized to the highest value of that fluorophore in each experiment.

### Adhesion and Spreading Assays

For adhesion and spreading assays with recombinant E-cadherin, glass bottom 24-well or 96-well plates were coated with goat anti-human IgG at 5 μg/mL and incubated overnight at 4°C. Non-adherent IgG was washed off with PBS + Ca^2+^, and Fc-tagged recombinant human E-cadherin (Acro Biosystems, Newark, DE) at 2 μg/mL or an equal volume human serum was added to wells and incubated at room temperature for 1 h. Alternatively, wells were coated directly with 0.2 μg/mL recombinant human E-cadherin (R&D systems, Minneapolis, MN) for 1 h. After coating, wells were washed, blocked with human serum, and washed again. MoDCs were incubated for 30 min with an anti-human CD103 neutralizing antibody (20 μg/mL), an appropriate isotype control antibody or were left untreated and then were added to the plates at 5 × 10^5^/mL. After incubation for 40 min at 37°C, non-adherent cells were gently washed off and cells were fixed with Cytofix/Cytoperm (BD biosciences, San Jose, CA). MoDCs were blocked with 10% goat serum and stained with HLA-DR FITC, a secondary IgG2a FITC to enhance signal, and DAPI to label cell nuclei. Wells were then imaged on an EVOS FL Cell imaging system and analyzed using ImageJ.

To quantify DC adhesion to gastrointestinal epithelial cells, HT-29 cells were cultured on 48 well plates for 3 days at a starting concentration of 3 × 10^5^ per well to obtain a 100% confluency for co-culture. MoDCs generated in medium alone or in the presence of RA were harvested and pre-treated with DC culture medium containing one of the following for 30 min at 37°C: 2 mM Mn^2+^; 1 mM EGTA; 2 mM Mn^2+^ and 1 mM EGTA; anti-human CD103 (2–20 μg/mL); or anti-human E-cadherin (5 μg/mL). MoDCs were next plated with the HT-29 monolayers at 2 × 10^5^ cells per well and incubated for 2 h at 37°C, with antibodies or other additives remaining in the culture media. Following incubation, non-adherent cells were gently washed off with media, and adherent MoDCs and HT-29 cells were harvested using 0.25% trypsin/1 mM EDTA (Millipore, Darmstadt, Germany). Cells were then stained with HLA-DR FITC antibody, counting beads were added, and recovered MoDCs were quantified by flow cytometry. Experiments with < 5% DC adhesion were excluded from the analyses, because it was difficult to interpret minor changes in adhesion when overall adhesion levels were extremely low. Experiments with low overall adhesion correlated neither with low DC CD103 or E-cadherin expression levels nor with a specific passage number or reduced E-cadherin expression of the HT-29 cells.

To quantify CD103 expression following MoDC HT-29 co-culture, a subset of MoDCs were incubated without HT-29 cells and subjected to the same 0.25% trypsin/1 mM EDTA treatment. MoDCs incubated alone and those co-cultured with HT-29 cells were then stained for CD103 expression and analyzed by flow cytometry.

### Statistical Analysis

Data were analyzed using GraphPad Prism 6.05. Results are presented as mean ± SEM. Differences between values were analyzed for statistical significance by the two-tailed Student's *t-*test or one- or two-way ANOVA with appropriate *post-hoc* analysis as indicated. Differences were considered significant at *P* < 0.05.

## Results

### Gastric Intraepithelial DCs Contain a Significant CD103-Expressing DC Subset

**Figure 1:**
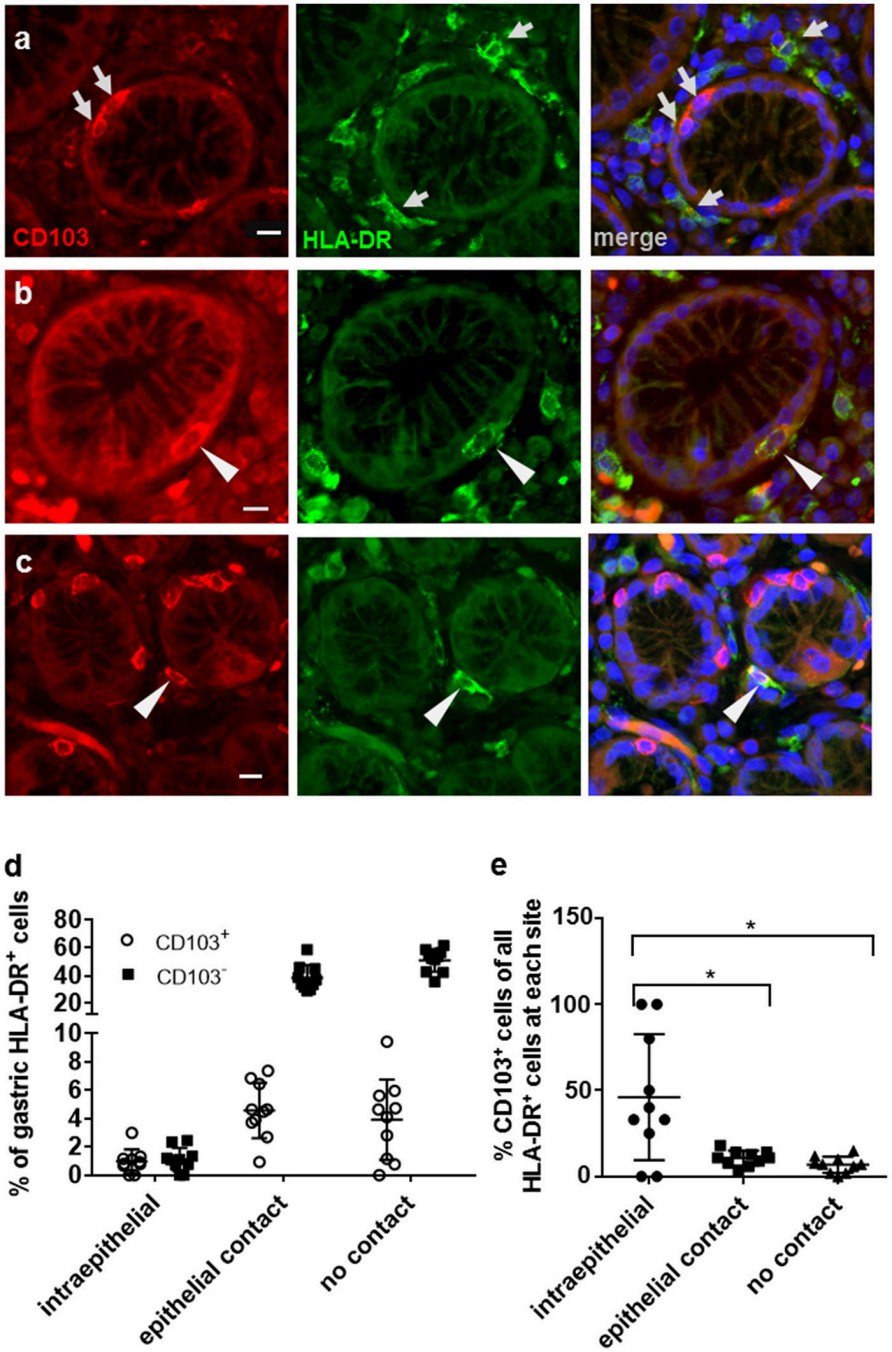
Distribution of CD103^+^ DCs in human gastric mucosa. Paraffin-embedded tissue sections from the gastric mucosa of non-*H. pylori*-infected human subjects were immunofluorescently labeled for HLA-DR (Alexa 488, green) and CD103 (Alexa 555, red). Nuclei were stained with DAPI. **(a)** High magnification single color and merged images of gastric mucosa with multiple cells positive for either CD103 or HLA-DR (arrows). **(b)** Occasional intraepithelial HLA-DR^+^ DCs and **(c)** subepithelial DCs with epithelial contact show staining for CD103. Arrowheads point out HLA-DR^+^ DCs that co-express CD103. Bar = 20 μm. **(d,e)** Quantitative analysis of HLA-DR^+^ DCs with and without CD103 expression at intraepithelial, subepithelial (with epithelial contact), and lamina propria sites (no epithelial contact). Tissue sections from 10 human subjects were analyzed. ^*^P ≤ 0.05, ANOVA with Tukey's *post-hoc* test.

Flow cytometric analyses of gastric DCs have shown that CD103^+^ DCs are rare in both human and murine stomach (14, 25–27). Here, we used immunofluorescence analysis of human gastric tissue sections to analyze CD103 expression by gastric mononuclear phagocytes in more detail. Mucosal DCs, and possibly some macrophages, were identified based on high expression of HLA-DR in conjunction with an irregular cell morphology. Notably, our previous studies showed that gastric HLA-DR^high^ cells are CD45^+^ leukocytes that express the DC-specific transcription factor zDC, but not the intestinal macrophage marker CD13 and that do not include B cells, T cells, mast cells, or NK cells (2, 14). Our immunofluorescence analyses confirmed that CD103 expression by HLA-DR^high^ cells in the gastric mucosa relatively rare (Figure 1a). Likewise, the majority of CD103^+^ cells, likely T lymphocytes, were negative for HLA-DR expression (Figure 1a). However, individual CD103^+^ HLA-DR^+^ DCs were detected in close association with the gastric glandular epithelium, either at intraepithelial sites, or directly below the epithelium (Figures 1b,c). We next performed a quantitative analysis of the distribution of CD103^−^ and CD103^+^ DC subsets in relationship to the gastric epithelium by counting DCs at intraepithelial, subepithelial (with epithelial contact), and lamina propria sites (no epithelial contact) (Figures 1d,e). Although intraepithelial HLA-DR^+^ DCs represented < 2% of all gastric mucosal DCs, a proportion (*P* ≤ 0.05) of these cells expressed CD103. Specifically, 46.1% of intraepithelial gastric HLA-DR^+^ cells were positive for CD103 compared to only 10.7 and 6.9% of CD103^+^ DCs at subepithelial and lamina propria sites, respectively (Figure 1e). These observations suggest that, in spite of an overall low expression of CD103 by human gastric DCs, CD103 might still contribute to DC interactions with the gastric epithelium in those DCs that are integrated into the epithelial cell layer.

### Intracellular Expression of CD103 (αE Integrin) in Human Monocyte-Derived and Gastric DCs

**Figure 2:**
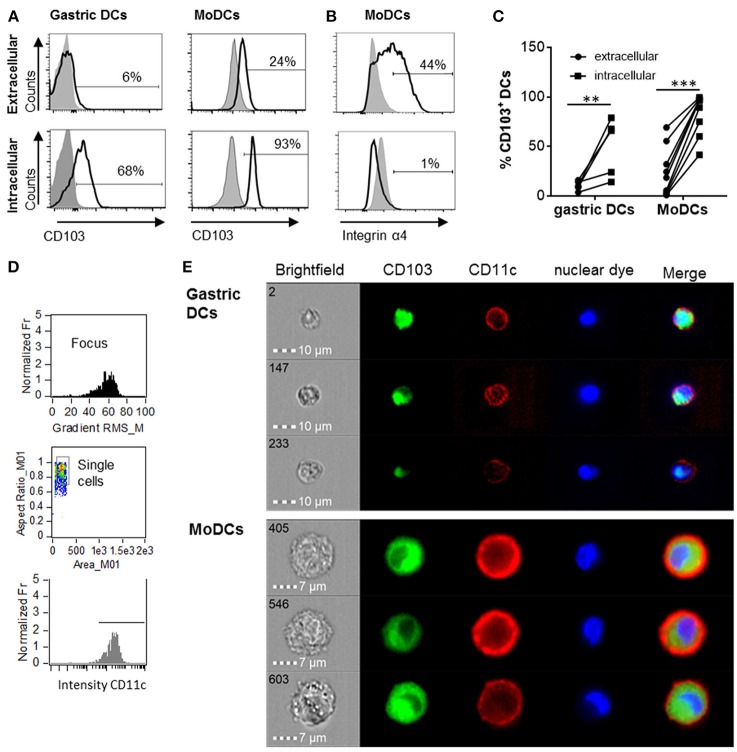
Human gastric and MoDCs contain intracellular CD103 pools. Human gastric DCs gated as live/HLA-DR^high^/CD45^pos^/lineage^neg^ cells or MoDCs were labeled with **(A)** an anti-CD103 antibody or **(B)** an anti-α4 integrin antibody using extracellular or intracellular staining protocols. **(A,B)** representative histograms and **(C)** individual values from 4 (gastric DCs) or 9 (MoDCs) independent experiments. ^**^P ≤ 0.01, ^***^P ≤ 0.001, ANOVA with Tukey's *post-hoc* test. **(D)** Gating strategy for ImageStream imaging cytometry to select for focused, single cells with high CD11c expression. **(E)** Representative images from three independent imaging cytometry experiments show intracellular CD103 in CD11c^+^ gastric DCs and MoDCs, 40 × objective. BF, bright field; nuclear dye, 7-AAD.

Human monocyte-derived DCs (MoDCs) contain intracellular as well as surface-expressed CD103 (26). Having shown that gastric DCs express low levels of surface CD103 overall (14, 26), but that surface CD103 expression is more frequent on gastric intraepithelial DCs (Figures 1b,e), we hypothesized that intracellular CD103 pools may be recruited to the cell membrane to mediate binding to epithelial E-cadherin. Therefore, we next analyzed whether primary human gastric DCs also express intracellular CD103. As shown in Figures 2A,C, significant levels of CD103 were detected in both gastric DCs and MoDCs when cells were permeabilized prior to immunolabeling, consistent with intracellular expression. In addition, intracellular CD103 expression was confirmed by imaging flow cytometry (Figures 2D,E). In contrast, we did not observe significant intracellular expression of α4 integrin, in spite of high surface expression (Figure 2B).

**Figure 3:**
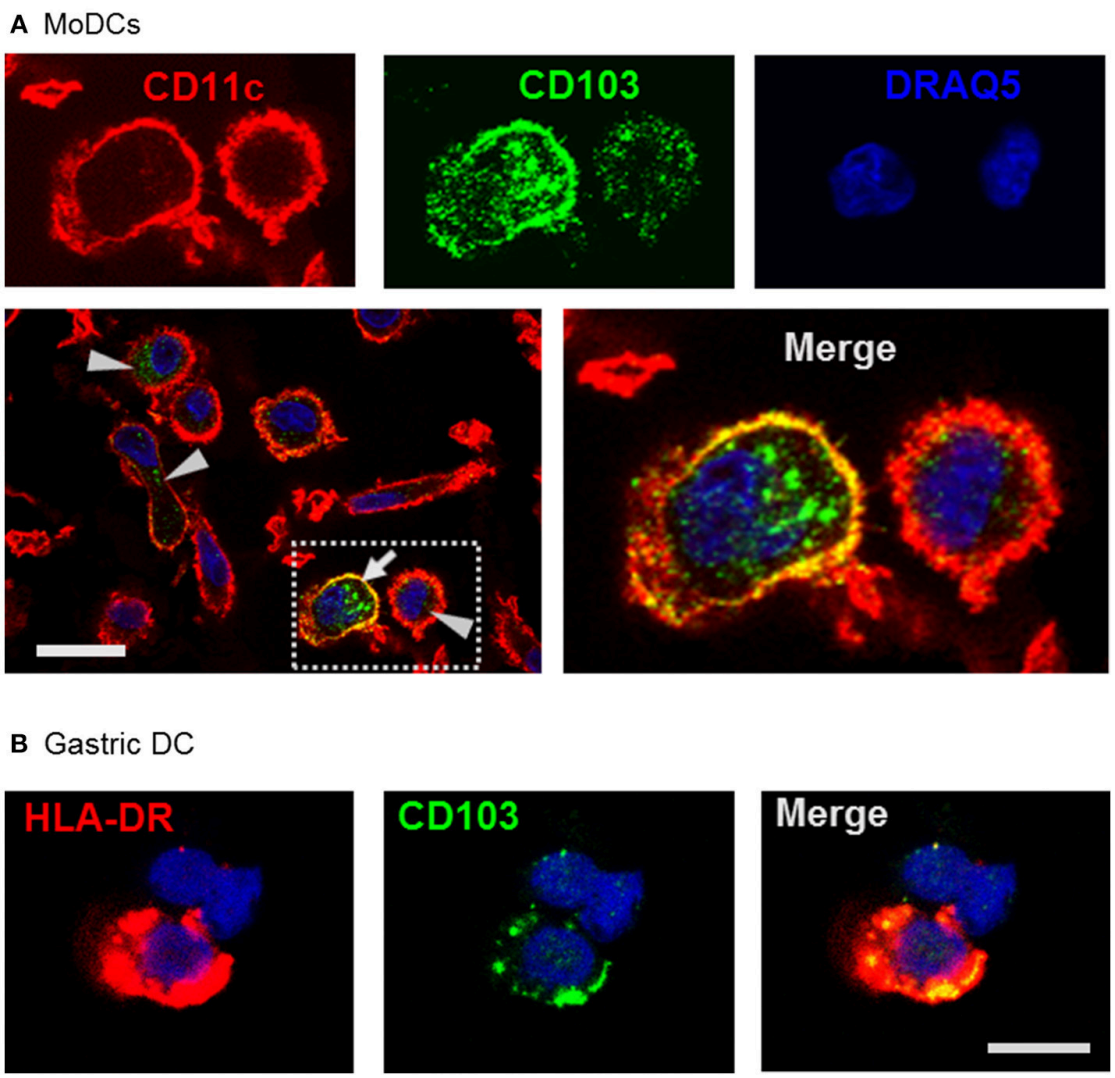
Confocal microscopy analysis shows endosomal expression pattern of CD103 in human monocyte-derived and gastric DCs. **(A)** Confocal images of human MoDCs that were permeabilized and labeled with antibodies to CD103 (Alexa488) and CD11c (Alexa555) Nuclei were labeled with DRAQ5. Arrowheads point to intracellular CD103^+^ vesicles in DCs without surface CD103 expression, arrow indicates a DC with both surface and intracellular CD103 expression. Representative images from one of three similar experiments are shown. Bar: 20 μm. **(B)** Gastric lamina propria cells were permeabilized and labeled with antibodies to CD103 (Alexa488) and HLA-DR (Alexa555). Image shows a gastric HLA-DR^+^ DC. Bar: 10 μm.

Using confocal microscopy, we detected CD103 in vesicular inclusions with a typical endosomal morphology in immature MoDCs with and without concurrent surface CD103 expression (Figure 3A). A similar vesicular staining pattern for CD103 was also seen in DCs isolated from human gastric lamina propria (Figure 3B). Thus, CD103 expression in endosomal compartments appears to be a common feature of human DCs.

### CD103 Partially Co-localizes With Clathrin and Early, Recycling, and Late Endosomal Markers

**Figure 4:**
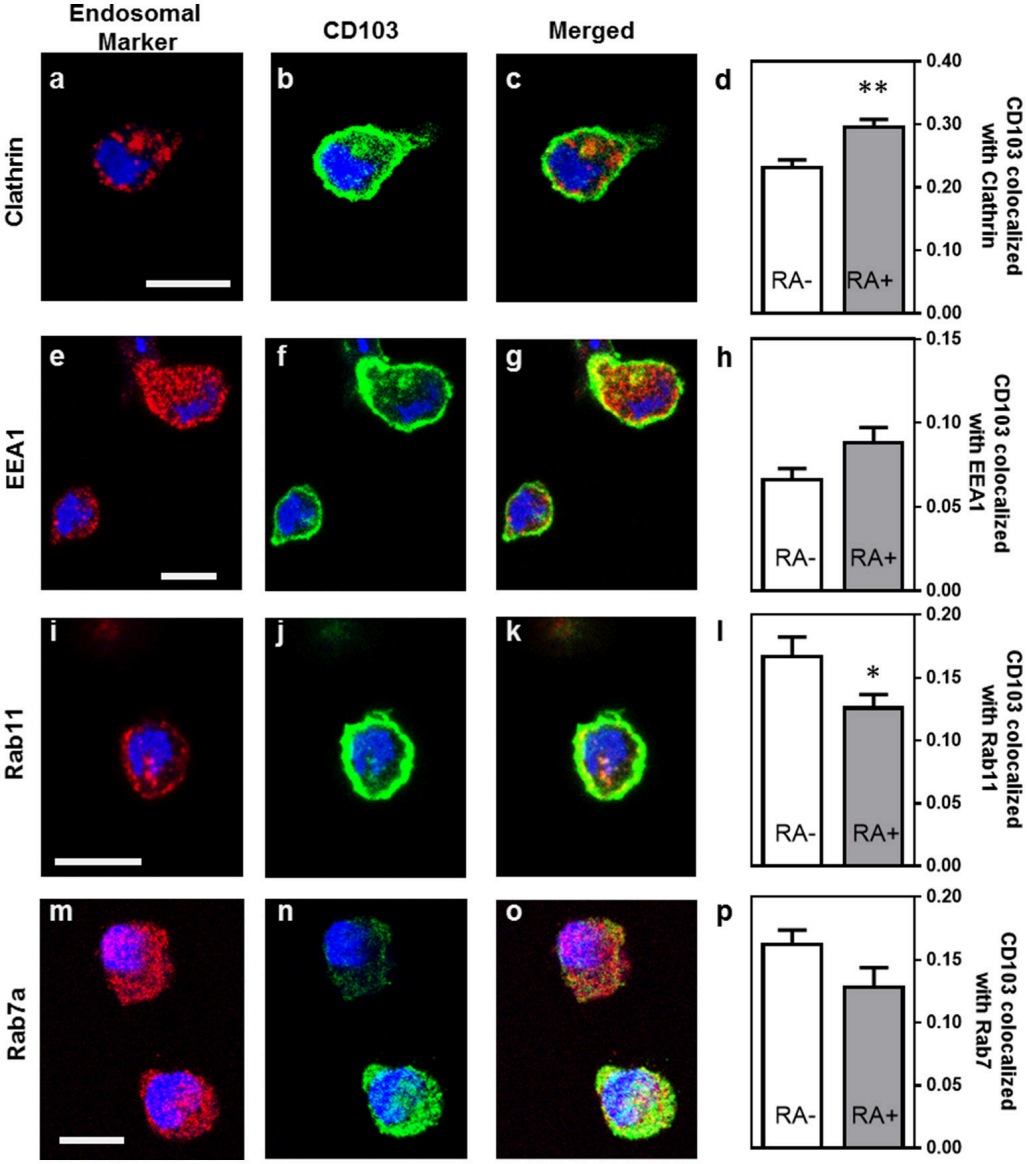
Partial co-localization of CD103 with endosomal markers in human MoDCs. MoDCs generated in medium alone or in the presence of retinoic acid (RA, 100 nM) were permeabilized and stained with antibodies to CD103 (green) and the following endosomal markers (red): **(a–c)** clathrin, **(e–g)** EEA-1, **(i–k)** Rab11, and **(m–o)** Rab7a. Images were obtained by confocal microscopy. Co-localization of CD103 with **(d)** clathrin, **(h)** EEA-1, **(l)** Rab11, and **(p)** Rab7a was calculated as the Manders' colocalization coefficient (M2) using ImageJ. Mean ± SEM of 10 −17 confocal images obtained from two independent experiments. Bar = 20 μm, ^*^P ≤ 0.05; ^**^P ≤ 0.01; unpaired Student's *T*-test.

Previous studies have shown that integrins may undergo endosomal trafficking to allow dynamic interactions with their ligands and facilitate cell migration (32, 37, 38). To characterize the endosomal expression of CD103 in human DCs in more detail, we analyzed co-localization of CD103 with markers for endocytic uptake (clathrin), early endosomes (early endosomal antigen-1, EEA-1), recycling endosomes (Rab11), and late endosomes (Rab7a) in MoDCs (Figure 4). In untreated MoDCs, 23.2 ± 1.2% of CD103 was co-localized with clathrin, corresponding to a Manders' co-localization coefficient of 0.232. Lower coefficients were detected for CD103 co-localization with Rab11 (16.7 ± 1.6%) and the late endosomal marker Rab7a (16.2 ± 1.2%), which targets endosomal cargo for lysosomal degradation (39). Only 6.6 ± 0.7% of CD103 co-localized with the early endosomal marker EEA-1. In control slides without primary antibodies, a co-localization co-efficient of 0.71 ± 0.69% was measured (data not shown). Interestingly, MoDCs treated with retinoic acid (RA) showed increased co-localization with clathrin (29.6 ± 1.3%; *P* ≤ 0.01), but lower co-localization with Rab11 (12.6 ± 1.1%; *P* ≤ 0.05). The partial co-localization of CD103 with endosomal markers suggests that CD103 undergoes some endosomal trafficking in human DCs.

### CD103 in Human MoDCs Undergoes Continuous Trafficking Through the Cell Membrane

**Figure 5:**
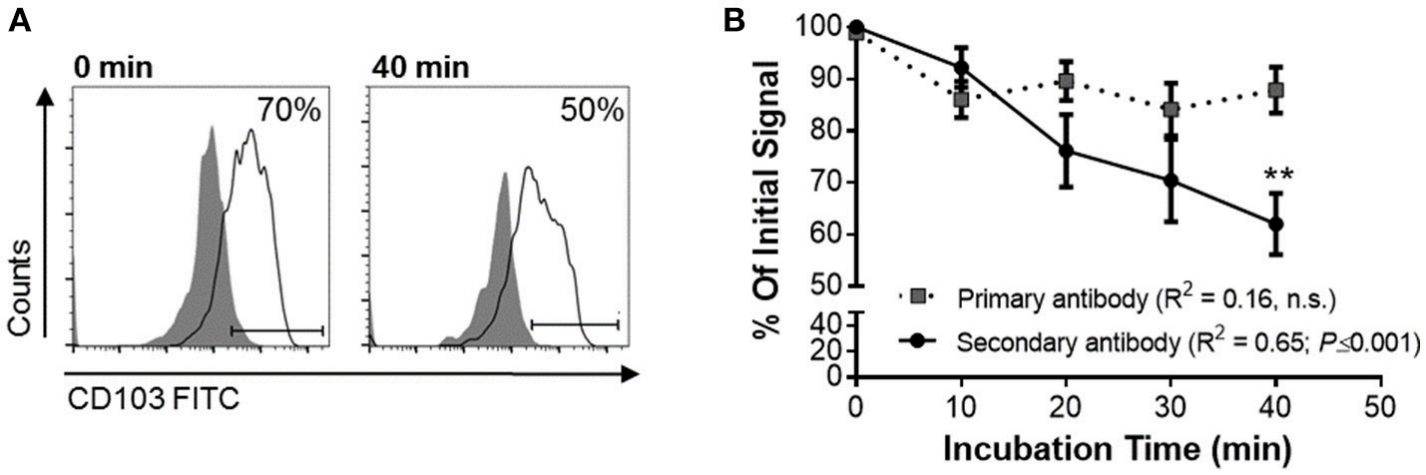
Internalization of surface-expressed CD103 in human MoDCs. Human MoDCs were labeled with an anti-CD103 FITC (mouse IgG1), washed, and chilled with ice-cold buffer. Time zero samples were left on ice, and internalization was initiated in the remaining samples by resuspending the samples in 37°C media and incubating them at that temperature for the indicated times. Following incubation, cells were harvested with cold buffer and then left on ice until all samples were collected. Anti-CD103 antibody remaining on the surface of the DCs was detected with a secondary anti-mouse IgG1 antibody. **(A)** Representative FACS plots and **(B)** pooled data from four independent experiments show a significant decrease in surface CD103 after 40 min. 2-way ANOVA with Sidak's multiple comparisons.

To functionally analyze whether DC CD103 undergoes endosomal recycling, we performed an internalization assay, as previously described by Chen et al. (36). RA-treated MoDCs were used to achieve a high initial expression of CD103 on the DCs. DCs were then incubated for up to 40 min at 37°C and were kept on ice at all other times to inhibit endosomal trafficking until all cells were collected, with the 0 min sample incubated on ice for the entire 40 min of the assay. As shown in Figure 5, the level of CD103 on the cell surface as detected with the secondary reagent decreased significantly with prolonged incubation at 37°C (*P* ≤ 0.01), whereas total CD103 expression detected with the primary antibody remained constant. These observations are consistent with internalization of surface expressed CD103 and indicate that CD103 undergoes endosomal recycling, as previously shown for α5β1 and α2β1 integrins (36, 37). Thus, CD103 may be involved in dynamic binding of DCs to the epithelial layer in spite of low surface expression.

### Intracellular CD103 Engages in E-cadherin Binding, but Does Not Mediate DC Adhesion to Epithelial Cells

**Figure 6:**
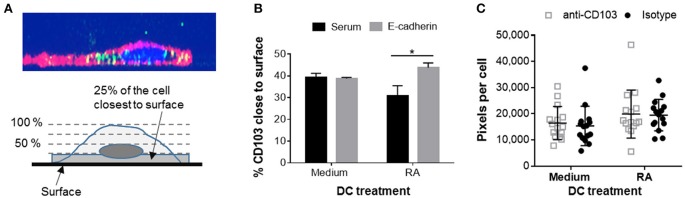
Adhesion of RA-treated MoDCs to E-cadherin-coated surfaces alters distribution of CD103. DC interactions with E-cadherin were analyzed by performing adhesion and spreading assays on glass slides coated with recombinant human E-cadherin. **(A,B)** RA-treated and untreated MoDCs were added to E-cadherin-coated slides and were incubated at 37°C for 40 min. Cells were then fixed, permeabilized and stained for CD103 expression. Z-stack images (0.5 μm step size) of adherent MoDCs were collected by confocal microscopy and analyzed for the distribution of CD103 in relation to the glass surface. **(A)** Top panel: orthogonal representation of an immunofluorescently labeled MoDC adhered to a glass surface and analyzed by z-stack confocal imaging. CD11c: red, CD103: green, DAPI nuclear stain: blue. Bottom panel: graphical representation of the image analysis approach. **(B)** Summarized data from three independent experiments were analyzed. ^*^P ≤ 0.05, 1-way ANOVA with Tukey's *post-hoc* test. **(C)** Spreading analysis of DCs on E-cadherin-coated glass slides. Glass slides were first coated with anti-human IgG antibodies and then with recombinant human E-cadherin-Fc. MoDCs generated in the presence of medium alone or 100 nM RA were pre-treated with a CD103 neutralizing antibody or an isotype control antibody for 30 min at 4°C. Cells were then added to the slides in the presence of 2 mM Mn^2+^ and incubated at 37°C for 40 min. Cell spreading was analyzed by fluorescence microscopy based on HLA-DR^+^ pixels per nucleus. Individual data points (*n* = 15 areas), mean and SEM from one representative out of 3 independent experiments are shown.

Having shown that human DCs contain dynamic pools of intracellular CD103, we next asked whether intracellular CD103 may be recruited to the DC surface for E-cadherin binding. RA-treated or untreated DCs were incubated on glass-bottom plates coated with recombinant E-cadherin (Supplemental Figure 1). The RA-treated DCs expressed increased levels of surface CD103 (Supplemental Figure 2). We hypothesized that engagement of E-cadherin by DC CD103 would lead to an accumulation of CD103 staining close to the E-cadherin-coated glass surface (Figure 6A). Our analysis of CD103 distribution across the vertical axis of the DCs showed a small, but significant shift in the proportion of CD103 close to the glass surface in RA-treated MoDCs in wells that were coated with E-cadherin compared to serum alone (Figure 6B, *P* ≤ 0.05). However, this trend was not observed in MoDCs generated in the absence of RA.

On hard surfaces, such as glass slides, adhesion complexes including integrin-dependent interactions influence cell spreading by enabling cells to extend actin-based lamellipodia (40). A previous study had shown that K562 cells transfected with αE(CD103)β7 formed epithelial protrusions and migrated on E-cadherin-coated surfaces (41). To determine whether CD103 promotes spreading and adhesion of human DCs on E-cadherin-positive surfaces, we used RA-treated and untreated MoDCs that were blocked with a CD103 neutralizing antibody or an isotype control antibody. As shown in Figure 6C, RA-treated DCs showed a trend (*P* = 0.05) for increased spreading on E-cadherin. However, DC spreading was not influenced by blocking CD103 on the DCs with a neutralizing antibody. The total number of adhered DCs also did not differ between treatments (data not shown). These results indicate that CD103 may relocate to the cell surface to engage in E-cadherin binding, but that overall DC adhesion to E-cadherin is largely independent of CD103.

### Bivalent Cations Promote DC Adhesion to E-cadherin-Expressing Gastrointestinal Epithelial Cells

**Figure 7:**
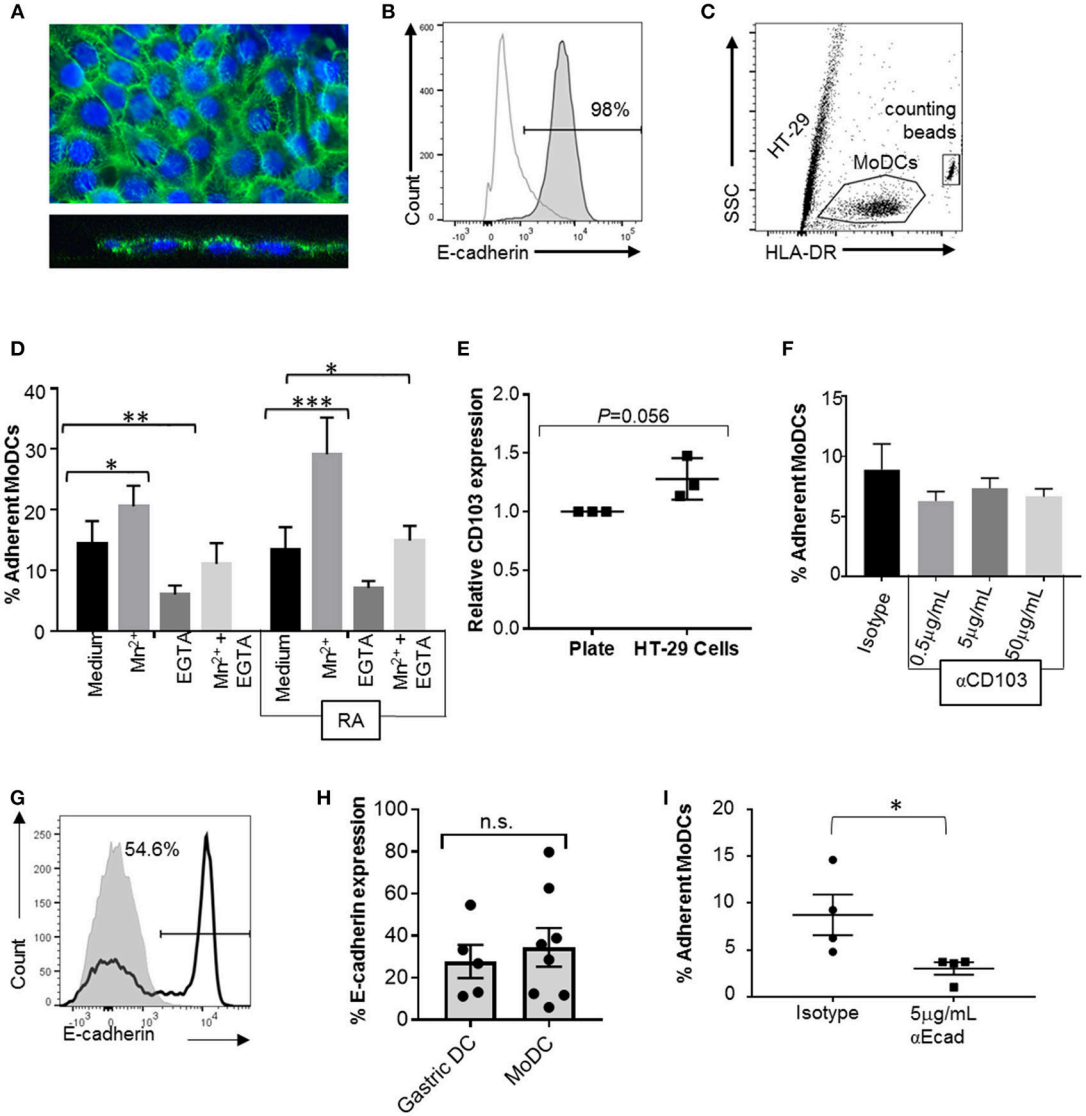
CD103 is not a major mediator of DC adhesion to E-cadherin-expressing epithelial cells. To analyze MoDC binding to E-cadherin expressed by epithelial cells, MoDCs were added to confluent monolayers of HT-29 cells for 2 h. Non-adherent cells were then removed by gentle washing, the remaining cells were collected by trypsinization, and the number of adherent DCs was determined using counting beads and HLA-DR-labeling of the DCs. **(A)** Confocal analysis of an HT-29 monolayer shows E-cadherin expression on the luminal surface. Top panel: maximum Z-projection; bottom panel: orthogonal view. Nuclei are labeled with DRAQ5 (blue). **(B)** Representative FACS histogram of E-cadherin expression on HT-29 cells. Gray line: isotype control; dark gray filled: anti-E-cadherin. **(C)** Gating strategy for counting adherent MoDCs following co-culture with HT-29 cells. **(D)** Percentage of MoDCs adherent to HT-29 cells in the presence of Mn^2+^, EGTA, or Mn^2+^ + EGTA (*n* = 6). RA indicates that cells were derived in the presence of 100 nM RA. ^*^P ≤ 0.05, ^**^P ≤ 0.01, ^***^P ≤ 0.001; 2-way ANOVA with Dunnett's multiple comparisons. **(E)** Normalized CD103 expression on MoDCs after recovery from HT-29 co-cultures (with Mn^2+^). Data from three independent experiments, paired, one-tailed Student's *t*-test. **(F)** MoDCs were incubated with an isotype control antibody or the indicated concentrations of anti-CD103 antibody before and during co-culture with HT-29 cells, and the number of adherent DCs was determined. *N* = 3, ^*^P ≤ 0.05; unpaired, two-tailed *T*-test. **(G,H)** representative histogram and pooled data for E-cadherin expression on human gastric (*n* = 5) and MoDCs (*n* = 8). **(I)** MoDCs were treated with anti-E-cadherin antibody before and during co-culture with HT-29 cells, and the number of adherent DCs was determined. *N* = 4, ^*^P ≤ 0.05; unpaired, two-tailed Student's *t*-test.

To analyze the interactions between MoDCs and cell-expressed E-cadherin, we performed MoDC adhesion assays with HT-29 cells, a colonic epithelial cell line strongly positive for E-cadherin. Importantly, HT-29 cells have an atypical E-cadherin distribution that involves E-cadherin expression on the apical cell surface (Figures 7A,B), but do not have any mutations in the E-cadherin gene *CDH1* (42, 43). DCs were incubated on top of HT-29 monolayers, and adherent cells were recovered by trypsinization after 2 h (Figure 7C). On average, 14.4 ± 3.7% of MoDCs were recovered from the cultures as adherent cells (Figure 7D). To determine whether integrins including αE integrin (CD103) are involved in DC adhesion to the HT-29 cells, we added 1 mM manganese (Mn^2+^), a strong activator of integrins that promotes ligand binding (44, 45). Both in RA-treated and untreated MoDCs, addition of Mn^2+^ significantly increased adhesion to HT-29 cells (*P* ≤ 0.001 and *P* ≤ 0.05, respectively). We also added EGTA, which inactivates bivalent cations, such as Ca^2+^, Mg^2+^, and Mn^2+^ that are involved in integrin- and E-cadherin-dependent interactions. EGTA significantly decreased the number of both and RA-treated and untreated DCs that were recovered from the co-cultures (*P* ≤ 0.05 and *P* ≤ 0.01, respectively). Although there was a trend for increased adhesion in RA-treated MoDCs, RA had no significant effect on DC binding to HT-29 cells, similar to our observations from the spreading analysis (Figure 6). Interestingly, co-culture with the HT-29 slightly increased surface expression of CD103 on the MoDCs (Figure 7E, *P* = 0.056).

### Neutralization of DC CD103 Does Not Inhibit Adhesion to E-cadherin Expressing Epithelial Cells

To specifically assess the involvement of CD103 in the interactions between DCs and epithelial cells, RA-treated MoDCs were blocked with a CD103 neutralizing antibody prior to adding the cells to the HT-29 monolayer in the presence of Mn^2+^. To avoid loss of blocking activity due to CD103 internalization, excess antibody was left in the cell culture medium during the adhesion assays. However, no decrease in DC adhesion to the HT-29 cells was seen with a wide range of antibody concentrations (Figure 7F).

### Homotypic E-cadherin Interactions May be Involved in DC Binding to E-cadherin on Gastrointestinal Epithelial Cells

To form adherens junctions, E-cadherin undergoes homotypic interactions with E-cadherin expressed on other cells (46), and DCs have been shown to express E-cadherin in previous studies (47–49). Our FACS analysis revealed that subsets of both human gastric DCs and MoDCs expressed E-cadherin (Figures 7G,H), independent of RA treatment (Supplemental Figure 2). Thus, E-cadherin-E-cadherin interactions may contribute to DC-epithelial cell interactions. Indeed, pre-treatment of the MoDCs with an E-cadherin neutralizing antibody significantly decreased DC adhesion to HT-29 monolayers (Figure 7I). However, when we compared MoDC adhesion to HT-29 cells with adhesion to AGS cells, which lack E-cadherin expression, we found that a significantly higher number of DCs adhered to the AGS cells than the HT-29 cells (Supplemental Figure 3, *P* ≤ 0.01). Interestingly, binding to AGS cells decreased when DCs were generated in the presence of RA. Overall, these data suggest that both E-cadherin-dependent and integrin-dependent mechanisms contribute to DC binding to the gastrointestinal epithelium, but that CD103-E-cadherin interactions are only minor contributors.

## Discussion

CD103 (αE integrin) is widely used as a maker for DC subsets in humans and mice (29), but the functional role of CD103 for the DCs has attracted little investigative attention. We here sought to elucidate whether CD103 could mediate DC-epithelial cell interactions in the human gastric mucosa. A number of previous reports had speculated that CD103 might mediate adhesion of gastrointestinal DCs to E-cadherin expressed in the epithelial layer (15–17), similar to the mechanism shown for the retention of intraepithelial lymphocytes within the epithelial compartment (22, 50). Studies from our laboratory have shown that DCs in the gastric mucosa are exposed to RA generated by gastric epithelial cells, and that RA induces CD103 expression in human MoDCs (14, 26). Our results from the present study suggest that CD103 is engaged upon binding of primary DCs to gastrointestinal epithelium, but is not a major mediator of adhesion.

One specific consideration when investigating CD103 in human gastric DCs was that < 10% of the DCs expressed CD103 on their surface, as we have previously shown (14, 26). However, based on the detection of intracellular CD103 (αE integrin), we hypothesized that these intracellular pools could be recruited to the cell surface for dynamic interactions with their ligands. Thus, integrins α5β1, α6β4, αMβ1, and α4β1 are continuously recycled through endosomal pathways during cell migration (33). However, not all integrins participate in the endocytotic cycle, and some integrins are recycled at only low rates (31). Indeed, we here confirmed that α4 integrin, which, like αE integrin, pairs with β7 integrin, was not expressed intracellularly. Our report is the first to demonstrate that CD103 in human DCs is expressed in endosomal compartments and recirculates through the cell membrane, suggesting that αE integrin recycling occurs in human DCs. It has been proposed that motile cells, such as DCs performing immunosurveillance functions, may require more trafficking of integrins and therefore contain a higher intracellular proportion (51). We demonstrated that 40% of surface CD103 was internalized in < 1 h. Moreover, 23–30% of CD103 co-localized with clathrin, consistent with the established role of clathrin in the endocytic recycling of integrin-mediated adhesions (52). A lower percentage of CD103 co-localized with the early endosome antigen 1 (EEA-1, 7%) and Rab11 (17%), a marker for long-loop endosomal recycling. Notably, previous publications have similarly reported co-localization co-efficients between 5 and 30% for integrins and endosomal markers in cells that were not specifically treated to enhance endosomal trafficking. Thus, Ezratty et al. (52) reported that 20–25% of β1 integrin co-localized with Rab5. In a publication by the Goldenring group, a Manders' co-localization co-efficient of >0.2 (>20%) for co-localization of Rab25 with β1 and α5 integrins was considered high (53). Gu et al. (54) from the Brenner lab analyzed co-localization of β3 integrin with endosomal markers and found between 2 and 8% of co-localization with EEA-1, Rab4, 5, and 11 at baseline, with increased co-localization upon PDGF-stimulated micropinocytosis. Khandelwal et al. (55) used a functional endocytosis assay with fluorescently labeled cargo and detected co-localization co-efficients of 10 and 20% for the endosomal cargo with EEA-1 and Rab11, respectively, and Karjalainen et al. (56) analyzed co-localization of α2 integrin with caveolin and detected 5–10% of co-localization at baseline. Therefore, we consider our observed co-localization of CD103 with the endosomal markers to be biologically relevant. Co-localization of CD103/integrin αE with clathrin, EEA1, and Rab11 suggests that integrin αE undergoes canonical trafficking similar to α5β1 integrin (57). However, the fact that CD103 also was co-localized with the late endosomal marker Rab7a may indicate that a proportion of intracellular CD103 is targeted for lysosomal degradation rather than recycling to the cell surface. Notably, when added together, < 60% of all CD103 co-localized with any endosomal marker, suggesting that a significant proportion of CD103 is present at sites that are not endosomal compartments. These might represent newly synthesized CD103 molecules in the endoplasmic reticulum or in the Golgi apparatus. Interestingly, RA treatment of the MoDCs led to increased co-localization with clathrin and EEA-1, but decreased co-localization with Rab11 and Rab7a. Thus, RA seems to both upregulate CD103 expression (26, 58) and alter its trafficking. Overall, our results suggest that, even on cells with low surface CD103 expression, CD103 may be recruited from endosomal pools for dynamic binding to epithelial E-cadherin or other ligands.

In support of a role for CD103 in human DC-epithelial interactions, we here showed significantly increased expression of CD103 on human HLA-DR^+^ DCs that were integrated into the gastric epithelial layer. Moreover, Z-stack analysis of MoDCs bound to E-cadherin-coated glass slides showed significant re-distribution of DC-expressed CD103 to the E-cadherin positive interface, albeit only in RA-treated cells. In addition, *in vitro* adhesion assays to E-cadherin-expressing HT-29 cells revealed a dependence on bivalent cations including manganese, consistent with an integrin-dependent mechanism (45). Conversely, when MoDCs were treated with a CD103 neutralizing antibody, adhesion to E-cadherin-positive HT-29 cells or to E-cadherin-coated glass slides was unaffected, arguing against a major role of CD103 in mediating DC binding to the gastrointestinal epithelium. Notably, while internalization of CD103 with bound blocking antibody may have decreased the efficiency of the neutralization, this would not be expected to completely abrogate functional activity of the blocking antibody, especially since a high antibody concentration (5 μg/mL) was used and additional antibody was present in the culture medium during the assay. Also, RA treatment, which increases MoDC CD103 expression, did not significantly influence MoDC binding to E-cadherin-expressing HT-29 cells or spreading on E-cadherin-coated glass surface, corroborating the results from the antibody neutralization experiments. Thus, adhesion of human DCs to gastrointestinal epithelia does not appear to be driven by CD103-E-cadherin interactions. Notably, we used high expression of HLA-DR to detect DCs in human gastric tissue sections, since no other more specific general DC marker has been identified for human stomach (2, 28). In the murine gastric mucosa, CX_3_CR1^+^ CD103^−^macrophages, and CD103^−^ DCs were able to sample *H. pylori* bacteria, whereas bacterial uptake by CD103^+^ DCs could not be detected (25), which also does not support our original hypothesis that CD103 positions gastric mononuclear cells at the epithelial interface for luminal *H. pylori* uptake. Along the same lines, an earlier report that investigated intraepithelial DCs in murine small intestine, the spatial relationship of murine intestinal DCs with the epithelium was not altered in CD103 knockout mice (59), whereas the number of IELs was significantly reduced in these animals (60). Together, these observations indicate that there are functional differences between T cell and DC-expressed CD103.

Our adhesion experiments did show that antibody inhibition of DC-expressed E-cadherin significantly suppressed DC binding to HT-29 cells, suggesting a role for homotypic E-cadherin-E-cadherin interactions. Thus, our results corroborate previous studies that showed Langerhans cells and other DCs of the skin and female genital tract interact with the epithelium through E-cadherin-E-cadherin binding (47, 61, 62). Notably, although homotypic E-cadherin interactions are calcium dependent, it appears that calcium can be replaced by the bivalent transitional elements manganese (Mn^2+^) and cadmium (Cd^2+^) (63), which would explain the observed increase in adhesion in the presence of Mn^2+^. The significant decrease in MoDC HT-29 adhesion in the presence of EGTA is consistent with either homotypic E-cadherin-E-cadherin interactions, which are calcium-dependent (64), or heterotypic E-cadherin interactions with αE integrin, which is activated by manganese (Mn^2+^) (65). Since E-cadherin is widely expressed on mucosal epithelial cells, it is a likely candidate for mediating the retention of motile immune cells at the epithelial barrier, and additional heterotypic E-cadherin ligands with expression on DCs including killer cell lectin-like receptor G1 (KLRG1) have been identified (22, 49, 66, 67). Surprisingly, MoDC adhesion to E-cadherin negative AGS cells was significantly higher than adhesion to HT-29 cells. These results suggest that DC adhesion to the gastrointestinal epithelium may involve additional molecular interactions independent of CD103 or E-cadherin, such as tight junction proteins (11, 68) and other integrins. Notably, all myeloid cells including DCs express β2 integrin (CD18). CD18 forms heterodimers with CD11a, CD11b, and CD11c (69) and contributes to cell-cell contact formation by binding to intracellular adhesion molecules (ICAMs), which may be expressed on gastric and intestinal epithelial cells (70, 71). Notably, adhesion of DCs to epithelial cells via β2 integrins would be consistent with our experimental observations that showed increased binding in the presence of Mn^2+^ and decreased binding in the presence of EGTA. Whether β2 integrins are major mediators of DC adhesion to the human gastric epithelium will be a subject of future studies.

If the interactions between DC-expressed CD103 and epithelial cells that we observed in human gastric tissue section do not result in strong adhesion, one might question the relevance of these interactions. However, engagement of CD103 by epithelial E-cadherin may lead to outside-in signaling through the cytoplasmic portion of the integrin (72). Previous studies have shown that in cytotoxic T cells, engagement of E-cadherin by CD103 triggers the phosphorylation of PLCγ1 and ERK1/2 (73), and engagement of CD103 by an anti-CD103 antibody can enhance T cell proliferation (74). Therefore, interactions between DC CD103 and epithelial E-cadherin could regulate certain DC functional characteristics through the activation of intracellular signaling cascades.

In summary, our study has provided novel insights in the regulation and function of CD103 (E integrin) in human DCs. We show that, like other integrins in motile cells, CD103 undergoes endosomal trafficking, which likely enables dynamic interactions between CD103 and its ligands. Our results also corroborate previous reports (59) that CD103 is not essential for the retention of DCs at gastrointestinal epithelial sites. The mechanisms by which CD103 on DCs in the human gastrointestinal tract interact with epithelial cells may be more subtle than simple adhesive interactions and requires further experimental exploration.

## Ethics Statement

This study was carried out in accordance with the recommendations of the U.S. Department of Health and Human Services' Policy for Protection of Human Research Subjects (45 CFR 46) with written informed consent from all subjects. All subjects gave written informed consent in accordance with the Declaration of Helsinki. The protocol was approved by the Institutional Review Boards of Montana State University and the University of Alabama at Birmingham.

## Author Contributions

DB and SS planned and oversaw the experiments. DB, SS, MR, BS, JD, TS, and RV performed the experiments. DB, PS, and LS initiated the project, critically discussed the data and obtained funding. DB, SS, and MR analyzed the data. MR, SS, and DB wrote the manuscript. All authors provided feedback on the manuscript.

### Conflict of Interest Statement

The authors declare that the research was conducted in the absence of any commercial or financial relationships that could be construed as a potential conflict of interest.

## Supplementary Material

Supplemental Figure 1 Coating of glass surfaces with rh E-cadherin. Untreated or poly-L lysine-coated wells of a glass bottom 96 well plate were incubated with recombinant human E-cadherin (1 μg/mL) in PBS for 60 min. Following a blocking step with 10% human serum, and several washes, E-cadherin bound to the plate was detected using an anti-E-cadherin antibody and visualized using an alkaline phosphatase detection system. Left panel: mean absorption (405 nm) ± SEM of duplicate wells. Right panel: plate image.

Supplemental Figure 2 Retinoic acid significantly increases expression of CD103, but not E-cadherin in human MoDCs. MoDCs were treated with 100 nM RA and surface expression of E-cadherin (left panel, *n* = 12) and CD103 (right panel, *n* = 13) were analyzed with flow cytometry. ^*^*P* ≤ 0.05; unpaired, two-tailed *T*-test.

Supplemental Figure 3 Strong adhesion of MoDCs to E-cadherin negative AGS cells. Top panel: confocal analysis of an AGS and an HT-29 monolayer shows surface E-cadherin expression (green) by the HT-29, but not by AGS cells. Bottom panel: RA-treated or untreated MoDCs were added to confluent monolayers of AGS or HT-29 cells for 2 h. Non-adherent cells were then removed by gentle washing, the remaining cells were collected by trypsinization, and the number of adherent DCs was determined using counting beads and CD11c-labeling of the DCs. Mean ± SEM of three independent experiments. Data were analyzed by ANOVA with Tukey's *post-hoc* test. a, b: different letters indicate significantly different values (*P* ≤ 0.05).
