# Supplementary figures and images for: CD103 (αE Integrin) Undergoes Endosomal Trafficking in Human Dendritic Cells, but Does Not Mediate Epithelial Adhesion
Source: Front Immunol. 2018 Dec 21;9:2989. doi: 10.3389/fimmu.2018.02989 (PMC6308147; doi:10.3389/fimmu.2018.02989)

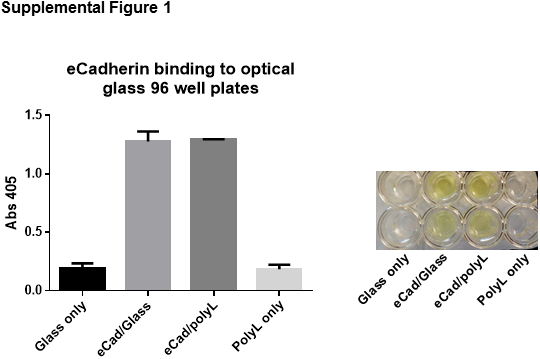

Supplement: Supplemental Figure 1 — Coating of glass surfaces with rh E-cadherin. Untreated or poly-L lysine-coated wells of a glass bottom 96 well plate were incubated with recombinant human E-cadherin (1 μg/mL) in PBS for 60 min. Following a blocking step with 10% human serum, and several washes, E-cadherin bound to the plate was detected using an anti-E-cadherin antibody and visualized using an alkaline phosphatase detection system. Left panel: mean absorption (405 nm) ± SEM of duplicate wells. Right panel: plate image. [file Image_1.TIF]

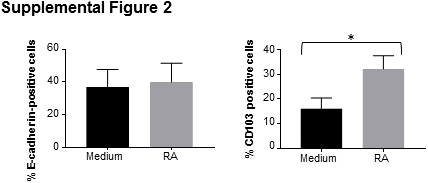

Supplement: Supplemental Figure 2 — Retinoic acid significantly increases expression of CD103, but not E-cadherin in human MoDCs. MoDCs were treated with 100 nM RA and surface expression of E-cadherin (left panel, n = 12) and CD103 (right panel, n = 13) were analyzed with flow cytometry. *P ≤ 0.05; unpaired, two-tailed T-test. [file Image_2.TIF]

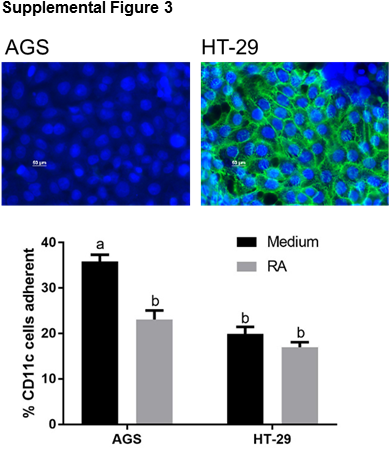

Supplement: Supplemental Figure 3 — Strong adhesion of MoDCs to E-cadherin negative AGS cells. Top panel: confocal analysis of an AGS and an HT-29 monolayer shows surface E-cadherin expression (green) by the HT-29, but not by AGS cells. Bottom panel: RA-treated or untreated MoDCs were added to confluent monolayers of AGS or HT-29 cells for 2 h. Non-adherent cells were then removed by gentle washing, the remaining cells were collected by trypsinization, and the number of adherent DCs was determined using counting beads and CD11c-labeling of the DCs. Mean ± SEM of three independent experiments. Data were analyzed by ANOVA with Tukey's post-hoc test. a, b: different letters indicate significantly different values (P ≤ 0.05). [file Image_3.TIF]
